# TSC1 Promotes B Cell Maturation but Is Dispensable for Germinal Center Formation

**DOI:** 10.1371/journal.pone.0127527

**Published:** 2015-05-22

**Authors:** Xinxin Ci, Masayuki Kuraoka, Hongxia Wang, Zachary Carico, Kristen Hopper, Jinwook Shin, Xuming Deng, Yirong Qiu, Shyam Unniraman, Garnett Kelsoe, Xiao-Ping Zhong

**Affiliations:** 1 Department of Pediatrics, Duke University Medical Center, Durham, NC, 27710, United States of America; 2 Key Laboratory of Zoonosis Ministry of Education, Institute of Zoonosis, College of Animal Science and Veterinary Medicine, Jilin University, Changchun, 130062, China; 3 Department of Immunology, Duke University Medical Center, Durham, NC, 27710, United States of America; 4 Laboratory Medicine Center, Nanfang Hospital, Southern Medical University, Guangzhou, Guangdong, 510515, China; University of Massachusetts Medical Center, UNITED STATES

## Abstract

Accumulating evidence indicates that the tuberous sclerosis complex 1 (TSC1), a tumor suppressor that acts by inhibiting mTOR signaling, plays an important role in the immune system. We report here that TSC1 differentially regulates mTOR complex 1 (mTORC1) and mTORC2/Akt signaling in B cells. TSC1 deficiency results in the accumulation of transitional-1 (T1) B cells and progressive losses of B cells as they mature beyond the T1 stage. Moreover, TSC1KO mice exhibit a mild defect in the serum antibody responses or rate of *Ig* class-switch recombination after immunization with a T-cell-dependent antigen. In contrast to a previous report, we demonstrate that both constitutive Peyer’s patch germinal centers (GCs) and immunization-induced splenic GCs are unimpaired in TSC1-deficient (TSC1KO) mice and that the ratio of GC B cells to total B cells is comparable in WT and TSC1KO mice. Together, our data demonstrate that TSC1 plays important roles for B cell development, but it is dispensable for GC formation and serum antibody responses.

## Introduction

In adult mice, B cells are generated in the bone marrow (BM). Following commitment of hematopoietic progenitors to the B-lineage differentiation, successive cellular events take place at distinct developmental stages defined as pro-B, pre-B, immature B, and transitional 1 (T1) B cells [1]. After maturation into the T1 stage, B cells emigrate from the BM to the spleen and mature further into T2 B cells and, eventually, enter into the long-lived mature B cells of the follicular (FO) and marginal zone (MZ) B cell compartments [2, 3]. The T2 B cells that successfully complete their maturation develop into either FO B cells or MZ B cells [4, 5]. B cells are agents of humoral immunity because they give rise to antibody-secreting plasma cells. During T-cell-dependent (TD) antibody responses, activated B cells form germinal centers (GCs) that are specialized structures within secondary lymphoid organs and are critical for the generation of B cell memory and high-affinity serum antibody responses [6]. In GCs, activated B cells proliferate and acquire high frequencies of point mutations in the rearranged V(D)J gene segments that constitute the immunoglobulin (Ig) variable (V) region; these *Ig* mutations are generated by the activation-induced cytidine deaminase (AID)-dependent process of somatic hypermutation (SHM) [7]. The GC microenvironment not only supports the expression of AID but also mediates the affinity-driven selection of mutant B cells [8, 9]. This selection process is necessary for the high-affinity memory B cell and antibody responses crucial for protection against microbial infection [6].

The mammalian target of rapamycin (mTOR) plays a critical role in activating cell-signaling pathways that regulate protein synthesis, metabolism, cell-cycle progression, cell growth, and cell proliferation. The mTOR signals are mediated by two complexes, mTOR complexes 1 and 2 (mTORC1/2). Both mTORC1 and mTORC2 are multimolecular complexes that share several common components, such as mammalian lethal with SEC13 protein 8 (mLST8) and DEP domain-containing mTOR-interacting protein (DEPTOR). In contrast, mTORC1 and mTORC2 contain unique components such as regulatory associated protein of mTOR (Raptor), and 40 kDa Pro-rich Akt substrate (PRAs40) for mTORC1, rapamycin-insensitive companion of mTOR (Rictor), mammalian stress-activated MAP kinase-interacting protein 1 (mSin1), and protein observed with Rictor1/2 (PROTOR1/2) for mTORC2. These different molecular compositions result in differences in the substrate selectivity and the biological processes regulated by each mTOR complex [10]. The mTORC1 phosphorylates pS6K1 and 4E-BP1 to increase ribosomogenesis and protein translation that are essential for cell growth and proliferation [11, 12]. The mTORC2 regulates cell survival and actin rearrangement by phosphorylating Akt at serine 473 and PKCα, respectively [13, 14]. Recent evidence has revealed the critical roles of mTOR activity for both innate and adaptive immune responses [15–17]. In T cells, mTOR promotes effector T-cell differentiation; inhibits inducible regulatory T-cell generation; controls CD8^+^ memory T-cell responses; and regulates T-cell trafficking, regulatory T-cell function, and iNKT cell maturation and function [18–24]. Despite extensive studies on T cells, the role of mTOR in B cells is poorly understood. A recent study found that mice with decreased mTOR activity manifest a partial block of B-cell development with lower numbers of pro-B, small and large pre-B, and mature B cells as well as reduced plasma cell numbers. Mature B cells with decreased mTOR activity exhibited impaired proliferation, antibody production, and chemotaxis [25]. An additional study demonstrated that mTORC2 is important for mature B-cell survival and proliferation [26].

The TSC1/2 complex, a heterodimer of TSC1 and TSC2, functions as a tumor suppressor by inhibiting mTORC1 [27]. The mTORC1 activation is dependent on the association of the GTP-bound active form of RheB (Ras homolog enriched in brain, a member of the small GTPase superfamily) with the complex. TSC2 inhibits RheB and, thus, mTORC1 via its GTPase activity [27–29]. Association of TSC2 with TSC1 is essential for TSC2 stability, and deletion of either TSC1 or TSC2 leads to enhanced mTORC1 signaling in cell lines and in primary immune cells [29–33]. Several studies have demonstrated that TSC1 plays critical roles in T cells, mast cells, and innate immunity by regulating mTOR signaling. Deficiency of TSC1 in T cells causes loss of T-cell quiescence and increased T-cell death due to the activation of the intrinsic death pathway, leading to reduced peripheral T-cell numbers [30, 34–36]. In addition, TSC1 plays important roles in conventional αβ T-cell and iNKT cell anergy [37–39], CD8 T-cell-mediated primary and memory responses [40], iNKT-cell terminal maturation and effector lineage fate decision [41], regulatory T-cell development and function [42, 43], endotoxin tolerance [31], antigen presentation by dendritic cells [44], mast cell degranulation and allergic responses [32], and hematopoietic stem cell quiescence [45]. A single study reported that TSC1 deficiency in B cells causes impaired B-cell maturation after the T2 stage, defective GC formation, and substantially decreased antibody responses to both TD and T-independent (TI) antigens [46]. Using B-cell-specific TSC1-deficient mice that have been backcrossed for eight to ten generations onto the C57BL/6J background, we have revisited the role of TSC1 in B cells. Consistent with the previous report, we observed abnormal B-cell development in TSC1-deficient (TSC1KO) mice, such as an overrepresentation of splenic T1 B cells and decreased FO and MZ B cells in the spleen. In contrast to the previous report, however, we observed less fitness of TSC1 KO T2 B cells compared to their WT counterparts. Despite impaired B-cell maturation, TSC1-deficient mice were competent to elicit the serum antibody and GC responses to immunization with TD antigen, NP-CGG precipitated in alum. These observations demonstrate that TSC1 plays a role in B-cell development at the transition from T1 to T2 B cells in a way that prevents the accumulation of T1 B cells. Our data also demonstrate that TSC1 is important for B-cell maturation/maintenance starting at the T2 stage as well as the FO and MZ B cell stages, but is dispensable for GC formation.

## Materials and Methods

### Mice

The *Tsc1* ^*flox/flox*^ mice [47] and *CD19-Cre* mice [48] were purchased from the Jackson Laboratory and Taconic Farm, respectively. *Tsc1* ^*f/f*^ mice were backcrossed to the C57BL/6 background for eight generations before being bred with the *Cd19-Cre* mice to generate *Tsc1* ^*f/f*^ *-Cd19Cre* ^+^ (TSC1KO or KO) and *Tsc1* ^*f/f*^-*Cd19Cre* ^-^ (WT) mice that were utilized to generate all the data except one experiment, which used the same mice in C57BL/6/129 mixed background. All TSC1KO mice used in this study were heterozygous for the *Cd19-Cre* targeted *Cd19* allele and were about 2 months of age. In addition, *Tsc1* was acutely deleted in *Tsc1* ^*f/f*^-*ERcre* mice [31, 49] after intraperitoneal injection of tamoxifen (2 mg/day) on days 1, 2, and 5, as previously described. Tamoxifen-treated mice were euthanized for isolation of splenic B cells on day 8. All mice were housed in a pathogen-free facility.

### Ethics Statement

This study was carried out in strict accordance with the recommendations in the *Guide for the Care and Use of Laboratory Animals of the National Institutes of Health*. All mice were used according to protocols approved by the Institutional Animal Care and Use Committee of Duke University.

### Total and subtype Ig quantification

Serum was collected from WT and TSC1KO mice at 2 months of age. Serum IgM and total IgG, IgG1, IgG2b, IgG2c, and IgG3 concentrations were determined by ELISA according to previously published protocols [50]. In brief, serum samples were initially diluted 1/500 with PBS, followed by serial 3-fold dilutions. Fifty microliters of diluted samples were added to 96-well plates precoated with anti-mouse Igκ and Igλ antibodies (2 μg/ml; Southern Biotech) in 0.1M carbonate buffer (pH 9.0) overnight at 4°C. Total and subtype Ig concentrations were determined using HRP-conjugated goat anti-mouse total or Ig subtype antibodies (Southern Biotech). The levels of these immunoglobulins were computed using an in-plate standard curve.

### Immunization and antibody responses

Mice were immunized by a single i.p. injection of 20 μg of 4-hydroxy-3-nitrophenylacetyl conjugated chicken gamma globulin (NP_11_-CGG, a TD antigen) in alum. Mice were euthanized on day 8, day 16, and day 24 following immunization for collection of the blood and spleen. To measure serum NIP-specific IgM and IgG levels, ELISA plates (Costar) were coated with 50 μl 2 μg/ml NIP_25_-BSA in 0.1 M carbonate buffer (pH 9.0) overnight at 4°C. Mouse sera were initially diluted 1:500, followed by 5–10 serial 3-fold dilutions. Fifty microliters of diluted samples were added to NIP_25_-BSA coated plates in duplicates. After incubation and multiple washes, NIP-specific IgM and IgG were detected by HRP-conjugated goat anti-mouse IgM and IgG, respectively. Purified mouse IgM (B1–8) and IgG (H33Lγ1) mAb were used to generate standard curves. All haptenated proteins were prepared using standard methods.

### Immunofluorescence studies

Peyer’s patches and spleens from nonimmunized and immunized mice were embedded in an optimal cutting temperature (OCT) compound (Sakura Finetek Inc., Torrance, CA), snap-frozen, and stored at −80°C. For germinal center identification, 5 μm thick frozen sections were stained with FITC-anti-GL-7, PE-anti-TCRβ, biotin-B220, and Alexa Fluor 350 Streptavidin. Images were acquired using a Zeiss Axiovert 200 M confocal immunofluorescent microscope.

### Flow cytometry

APC-, APC-Cy7, FITC-, PE, PE-Cy5, PE-Cy7, and biotin-conjugated antibodies specific for mouse CD93, B220, IgD, IgM, GL-7, Fas, CD21, CD23, and CD43 were purchased from Biolegend, eBioscience, or BD Biosciences. Single-cell suspensions from Peyer’s patch, BM, and the spleen were blocked with rat IgG (100 μg/ml; Sigma) and anti-mouse CD16/CD32 (5 μg/ml; BD Biosciences) and then stained with fluorochrome-conjugated antibodies. PI was used for the exclusion of dead cells. Stained cells were collected using a BD CantoII flow cytometer (BD) and analyzed using FlowJo software (Tree Star).

### Bone marrow chimeras

*Rag-1* ^*-/-*^ mice were sublethally irradiated with 600 rad of γ-ray and reconstituted with equal numbers of BM cells (10 × 10^6^ each) from CD45.1^+^ C57BL/6 mice and CD45.2^+^ TSC1KO mice via retro-orbital eye injection. Eight weeks after reconstitution, chimeric mice were euthanized for assessment of B-cell development using flow cytometry.

### *In vitro* Ig class-switch

Spleens were harvested on day 8 from *Tsc1* ^*f/f*^-*ERcre* ^-^ and *Tsc1* ^*f/f*^-*ERcre* ^+^ mice after being injected with 2 mg tamoxifen on days 1, 2, and 5. B cells were prepared from mouse spleen by RBC lysis with ACK buffer and by enrichment with the EasySep B cell negative selection kit (Stem Cell Technologies). Purified B cells were cultured at 0.25 × 10^6^ cells/mL in RPMI-1640 supplemented with 10% FCS, 10 mM HEPES, 60 μg/mL penicillin, 100 μg/mL streptomycin, 2 mM L-glutamine, 1 mM sodium pyruvate, 0.1 mM nonessential amino acids, and 55 μM β-mercaptoethanol. Cells were induced to switch to different isotypes under the following conditions: IgG1 (10 μg/mL LPS (Sigma-Aldrich), 10 ng/mL rmIL-4 (R&D Systems), 50 ng/mL BAFF (Peprotech), IgG3 (10 μg/mL LPS), and IgA (5 μg/mL LPS, 1 ng/mL rhTGFβ (R&D Systems), and 50 ng/mL BAFF). After 4 days, cells were stained for a pan-B cell marker B220 or CD19 and IgG1 (BD), IgG3 (BD), or IgA (BD).

### Immunoblot

To isolate mature B cells, they were first enriched from splenocytes by negative selection using a cocktail of anti-CD138, TER-119, Fas, CD11b, GL-7, Gr-1, Thy1.2, CD5, and CD43 antibodies. Enriched B cells were stained with anti-B220, anti-CD93, and propidium iodide (PI) and then sorted for live CD93^-^B220^high^ mature B cells. To isolate GC B cells, WT and TSC1KO mice were immunized with NP_11_-CGG. Eight days after immunization, splenocytes from immunized mice were first enriched by positive selection using an anti-Fas antibody and MACS column. Enriched cells were stained with anti-B220, anti-CD93, anti-GL7, and 7AAD and then sorted for live CD93^-^B220^high^ Fas^+^GL7^+^ GC B cells. To isolate B cells from tamoxifen-treated *Tsc1* ^*f/f*^-*ERcre* mice, splenocytes from these mice and control mice were stained with anti-B220 and positively selected with MACS columns followed by 7AAD staining and FACS sorting for B220^+^ cells. Sorted B cells were lysed in 1% nonidet P-40 lysis buffer (1% NP-40, 150 mM Nacl, 50 mM Tris, pH 7.4) with freshly added protease and phosphatase inhibitors. Cell lysates were subjected to immunoblotting analysis using the following antibodies: anti-TSC1, anti-phospho-p70S6K T421/424, anti-p70S6K, anti-phospho-4E-BP1 T37/S46, anti-4E-BP1, anti-phospho-Akt S473, anti-Akt, anti-phospho-Foxo1 S256 (all from Cell Signaling Technology), and anti-β-actin (Sigma-Aldrich). The membranes were further probed with HRP-conjugated secondary antibodies (Bio-Rad) and detected by ECL Western Blot Substrate (PerkinElmer).

### Real-time quantitative PCR

Genomic DNA from indicated B-cell subsets sorted from *Tsc1* ^*flox/flox*^ mice and *Tsc1* ^*flox/flox*^-*Cd19-Cre* mice using MoFlo Cell Sorter (Becman Coulter) was isolated using a standard protocol. TSC1 deletion efficiency was quantified by real-time quantitative PCR (Mastercycler realplex, Eppendorf) using a pair of primers flanking a loxP site (5’-GTCACGACCGTAGGAGAAGC-3’ and 5’-GAATCAACC CCACAGAGCAT-3’) and SYBO green. Only undeleted *TSC1* alleles could be amplified by these primers. Data were calculated using the 2^ΔΔ^Ct method after normalization to genomic DNA of the *CD14* gene using *CD14* specific primers (5’-TCCATGCCCTGAAGTCATCCT-3’ and 5’-TGGGAACACGTCTCTGCACTT-3’).

### Statistical analysis

Two-tailed unpaired Student’s *t* tests were performed to determine *p* values. All the graphs represent mean ± SEM, and asterisks represent p values: *p < 0.05, **p < 0.01, ***p < 0.001.

## Results

### TSC1 deficiency in B cells caused developmental blockade at T1 stage

**Fig 1:**
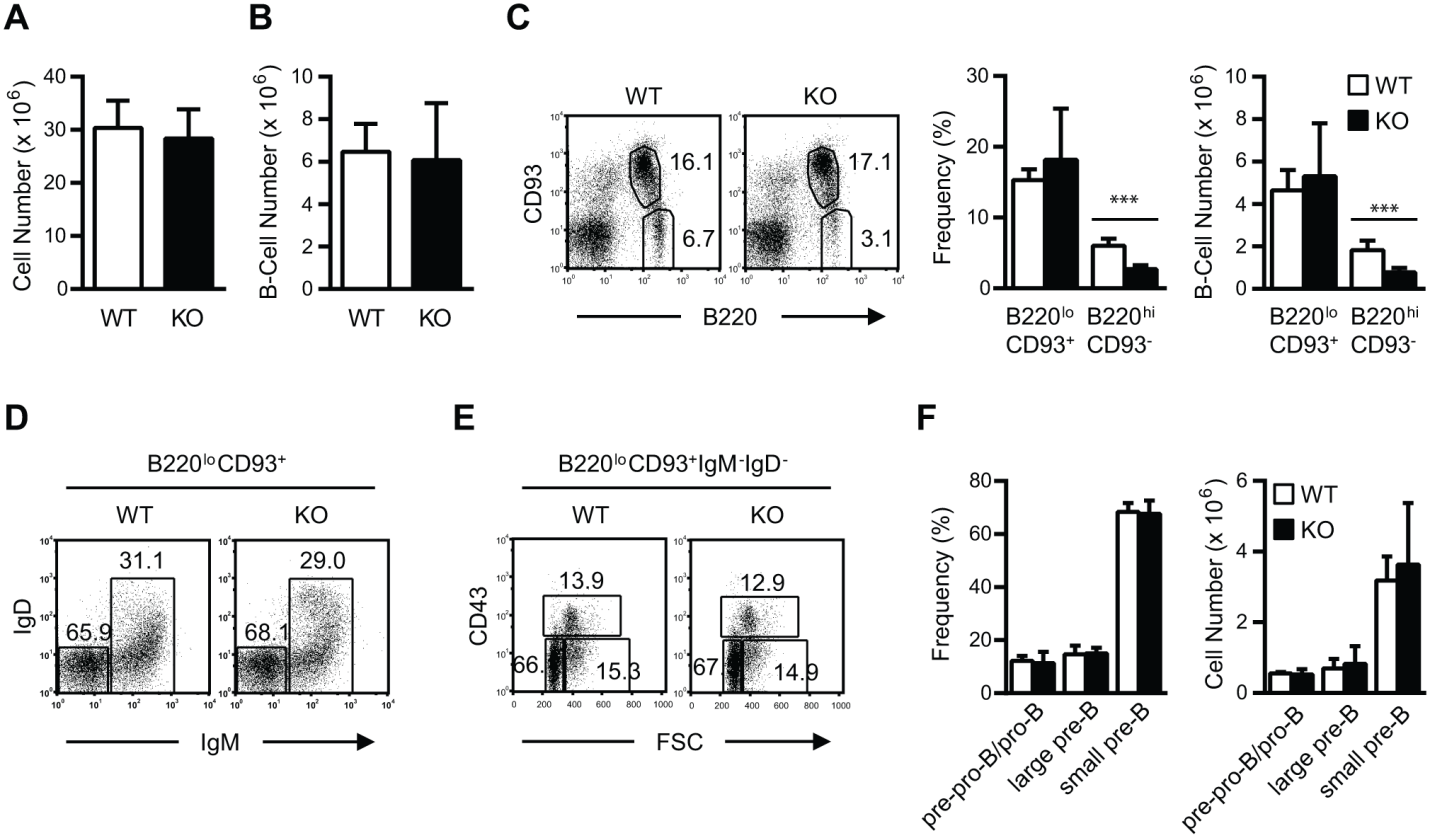
Effect of TSC1 deficiency on B cell development in the bone marrow. (A, B) Two-month-old WT and TSC1KO mice were euthanized to assess B cell development. BM total cellularity (A) and total B220^+^ B cells (B) (n = 8). (C) Decreased B220^high^CD93^-^ B cells in TSC1KO BM. Dot plots show representative CD93 and B220 staining of live-gated BM cells. Bar graphs represent mean ± SEM of percentages (middle) and absolute numbers (right) of indicated B cell compartments (n = 8). (D-F) Normal early B cell development in TSC1KO mice. (D) IgM and IgD staining of gated CD93^+^B220^low^ cells from BM. (E) CD43 staining and FSC assessment of gated CD93^+^B220^low^IgM^-^IgD^-^ BM cells. (F) Frequencies (% of CD93^+^B220^low^IgM^-^IgD^-^ B cells) and absolute numbers of pre-pro-B/pro-B, large pre-B, and small pre-B cells. Pre-pro-B/pro-B cells, CD93^+^B220^low^IgM^-^IgD^-^CD43^+^ FSC^int^; large pre-B, CD93^+^B220^low^IgM^-^IgD^-^CD43^int^FSC^high^; small pre-B, CD93^+^B220^low^IgM^-^IgD^-^CD43^-^FSC^low^ (n = 8). Data shown represent or are calculated from at least four experiments. Two pairs of WT and TSC1KO mice received BrdU prior to analysis. ***p<0.001 determined by Students’ *t* test.

To investigate the role of TSC1 in B cells, we backcrossed *Tsc1* ^*f/f*^ mice to the C57BL6/J background for eight generations; these backcrossed mice were subsequently bred with congenic *Cd19Cre* mice to generate *Tsc1* ^*f/f*^-*Cd19Cre* ^-^ (WT) and *Tsc1* ^*f/f*^-*Cd19Cre* ^+^ (TSC1KO) mice. TSC1KO and WT mice contained similar numbers of BM cells (Fig 1A), and the numbers of B220^+^ BM B cells were decreased only slightly in TSC1KO animals (Fig 1B). Similarly, the frequency and numbers of B220^low^CD93^+^ developing B cells were nonsignificantly reduced in TSC1KO mice (Fig 1C). Further analysis of the BM B220^low^CD93^+^ developing B cells showed that pre-pro-B/pro-B (CD93^+^B220^low^CD43^+^IgM^-^IgD^-^FSC^int^), large pre-B (CD93^+^B220^low^CD43^int^IgM^-^IgD^-^FSC^high^), and small pre-B (CD93^+^B220^low^CD43^-^IgM^-^IgD^-^FSC^low^) cells were comparable between TSC1KO mice and WT controls (Fig 1D–1F). In contrast, both the percentage and absolute number of B220^high^CD93^-^ mature B cells were decreased by about half in the BM of TSC1KO mice (Fig 1C).

**Fig 2:**
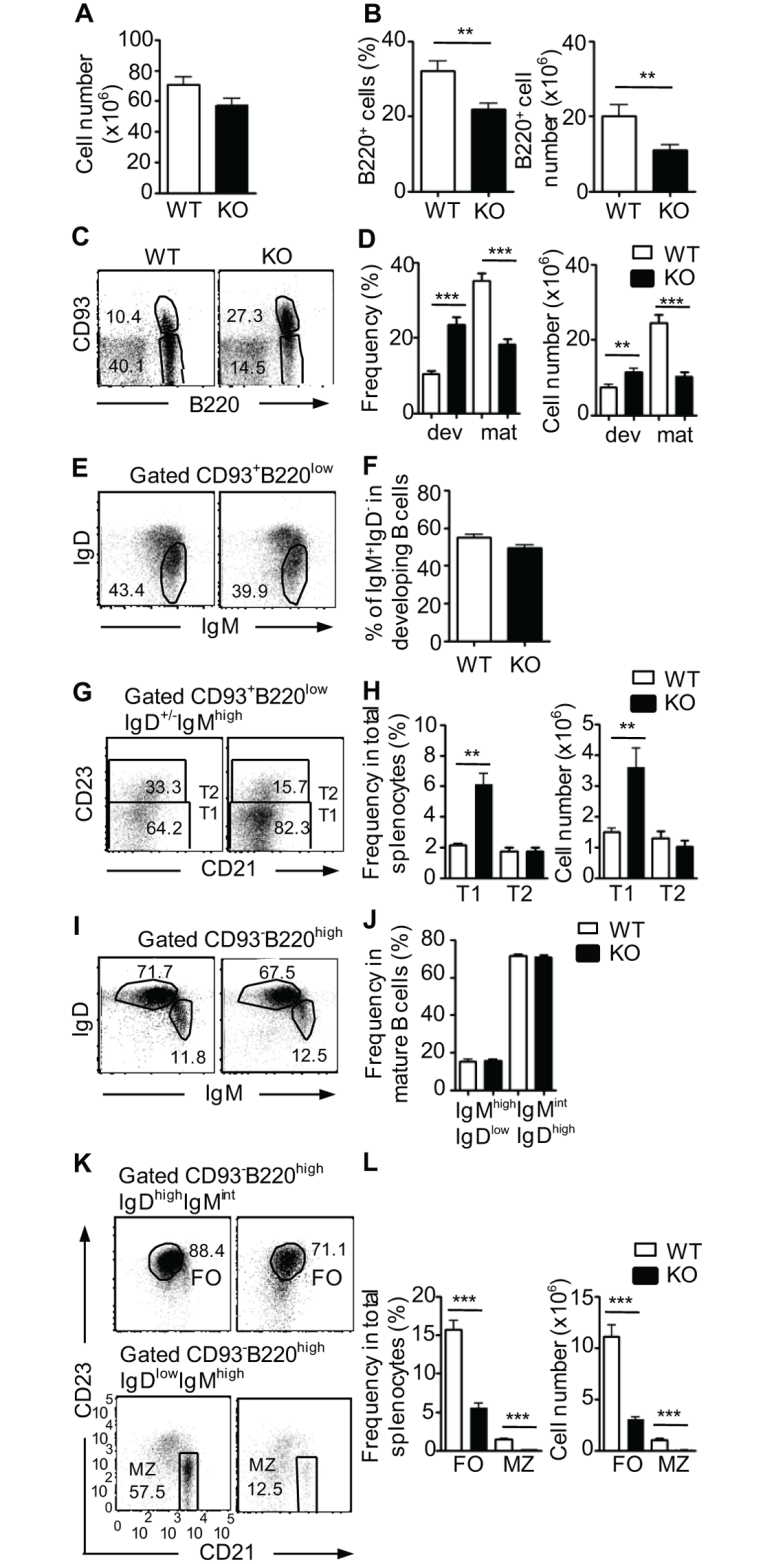
Effect of TSC1 deficiency on B cell development in the spleen. (A) Total splenocyte number in WT and TSC1KO mice. (B) Percentage (left panel) and total B cell numbers (right panel) in WT and TSC1KO spleens (n = 10). (C, D) Decreased mature but increased immature B cells in TSC1KO spleen. Dot plots show CD93 and B220 staining of splenocytes (C). Bar graphs show percentage (left panel) and absolute numbers (right panel) in WT and TSC1KO spleens (D). (E, F) IgM and IgD expression in CD93^+^B220^low^ compartment. (G, H) Increased T1 B cells in TSC1KO spleen. Dot plots show CD21 and CD23 expression in gated CD93^+^B220^low^IgM^high^IgD^+/-^ cells (G). Bar graphs show percentages and absolute numbers of T1 and T2 B cells (H). (I, J) IgM and IgD expression in CD93^-^B220^high^ cells. (K, L) decreased mature B cells in TSC1KO spleen. Dot plots show CD23 and CD21 expression to identify FO and MZ B cells in gated CD93^-^B220^high^IgM^int^IgD^high^ cells and CD93^-^B220^high^IgM^high^IgD^low^ cells, respectively (K). Bar graphs shows percentages and absolute numbers of FO and MZ B cells in the spleen (L). Data shown represent or are calculated from at least five experiments with a total of ten mice in each group. **p<0.01; ***p<0.001.

In the spleen, total cell numbers were similar in WT and TSC1KO mice (Fig 2A); however, both numbers and frequency of total B220^+^ B cells were decreased in TSC1KO mice (Fig 2B). In contrast to the reduction of total B cells, CD93^+^B220^low^ developing B cells were increased ~2-fold in TSC1KO mice compared to WT mice (Fig 2C and 2D). Within the CD93^+^B220^low^ developing B cells, the percentage of IgM^high^IgD^+/-^ T1/T2 B-cell compartments was similar between WT and TSC1KO mice (Fig 2E and 2F). Further analysis of CD21 and CD23 expression in CD93^+^B220^low^IgM^high^IgD^+/-^ T1/T2 B cells showed that both percentage and absolute number of CD21^-^CD23^-^IgM^high^IgD^+/-^ T1 B cells were elevated in TSC1KO spleens compared with WT controls (Fig 2G and 2H). In contrast, the percentage and absolute number of CD21^low^CD23^+^IgM^high^IgD^+/-^ T2 B cells in TSC1KO spleens were comparable to WT controls and were not concurrently increased with T1 B cells.

Similar to the BM, CD93^-^B220^high^ mature B cells were decreased in TSC1KO spleens (Fig 2C and 2D). Although the B220^high^CD93^-^ mature B cells were substantially decreased in TSC1KO spleens, the pattern and proportion of IgM/IgD expressions in mature B cells were similar between WT and TSC1KO mice (Fig 2I and 2J). Correlated with decreases of mature B cells, both IgM^high^IgD^low^CD23^low^CD21^high^ marginal zone (MZ) B cells and IgM^int^IgD^high^CD23^high^CD21^low^ follicular (FO) B cells were decreased in TSC1KO spleens, compared with WT controls (Fig 2K and 2L). Together, our data suggest that TSC1 is required for the transition from T1 B cells to T2 B cells, and is important for the generation and/or maintenance of mature B cells.

### TSC1 deficiency causes cell intrinsic accumulation of T1 B cells

**Fig 3:**
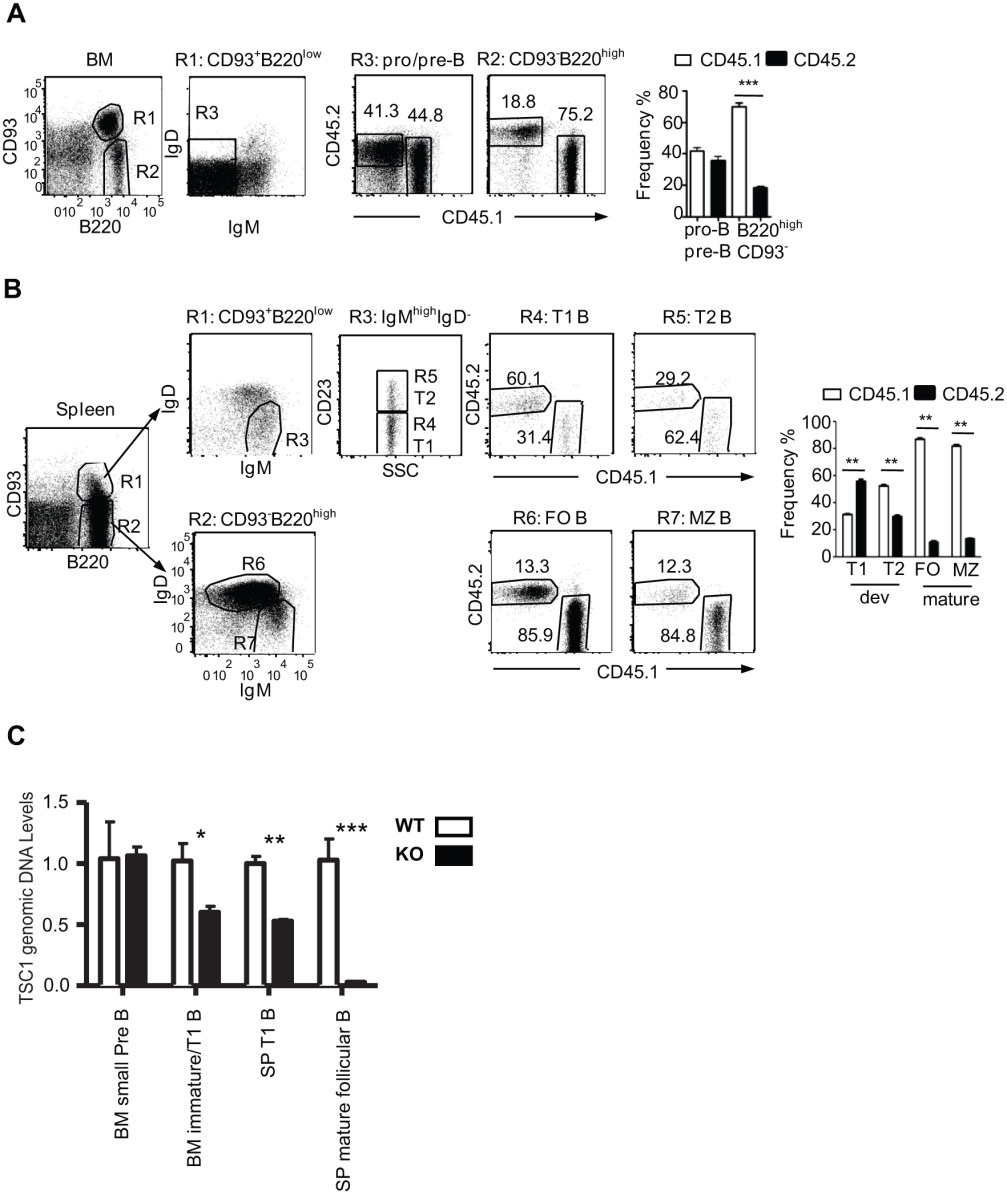
Accumulation of T1 B cells in TSC1KO mice is cell intrinsic. Sublethally irradiated *Rag-1*-deficient mice were reconstituted with mixed CD45.1^+^ WT and CD45.2^+^ TSC1KO BM cells at 1:1 ratio. Recipient mice were analyzed similar to the method shown in Figs 1 and 2, two months after reconstitution. (A) Assessment of B cell development in the BM. Developing B cells (R1) and mature B cells (R2) in the BM were identified by B220 and CD93 expression. IgM^-^IgD^-^ cells (R3) from R1-gated cells were further analyzed for CD45.1 and CD45.2 expression. Bar graph shows mean ± SEM of percentages of CD45.1 and CD45.2 positive cells in the indicated populations. (B) Assessment of B cell development in the spleen. Splenic developing (R1) and mature (R2) B cell compartments were identified by B220 and CD93 expression. T1/T2 B cells identified by R1 and R3 gating were further divided into CD45.1^+^ and CD45.2^+^ populations (top panels). Mature B cell compartment (R2) was further divided by IgM/IgD expression into mature follicular (R6) and marginal zone (R7) B cell compartments. CD45.1/CD45.2 expressions in each compartment are shown. Bar graphs are mean ± SEM of CD45.1 and CD45.2 percentages in the indicated populations (n = 4). (C) Assessment of *TSC1* deletion in B cell subsets. Genomic DNA from sorted B cell subsets was used to quantify *TSC1* gene in WT configuration by real-time qPCR. Bar graphs represent relative genomic DNA levels of undeleted *TSC1* gene. Data shown represent or are calculated from two experiments. *p<0.01; **p<0.01; ***p<0.001.

The accumulation of T1 B cells and the decrease of mature B cells in TSC1KO mice could be caused by multiple mechanisms. To determine whether each of the observed developmental abnormalities in TSC1KO mice is cell autonomous, we generated and analyzed mixed BM chimeric mice by injecting WT (CD45.1) and TSC1KO (CD45.2) B-cell-depleted BM at a 1:1 ratio into sublethally irradiated Rag-1-deficient mice. Eight weeks after reconstitution, both BM and spleens were harvested to assess B-cell development in these chimeric mice. An almost equal representation of CD45.1^+^ WT- and CD45.2^+^ TSC1KO-derived cells in BM CD93^+^B220^low^IgM^-^IgD^-^ (pro/pre B) compartment suggests equal contribution of WT and TSC1KO BM hematopoietic stem cells in the generation of B-cell progenitors in chimeric mice (Fig 3A). In contrast, TSC1KO-originated cells accounted for only 1/5 of CD93^-^B220^high^ mature B-cell compartments in the BM of chimeric mice (Fig 3A). In the spleen, TSC1KO-derived T1 B cells were twice as numerous as WT-derived T1 B cells. However, TSC1-derived T2 B cells accounted for only 1/3 of T2 B cells. The underrepresentation of TSC1KO B cells was even more severe in the mature FO and MZ B cell compartments, where TSC1KO cells accounted for only about 1/8 of the FO and MZ B cell compartments (Fig 3B). These observations demonstrate that cell-intrinsic mechanisms caused the accumulation of T1 B cells and the decrease of mature B cells in the absence of TSC1. Given that TSC1KO T2 B cells are less competent than their WT counterparts, our results strongly suggest that TSC1 also plays a role in T1 B to T2 B maturation.

It is noteworthy that CD19 expression begins at the pro-B cell stage during B-cell development [1], but the *Tsc1* gene appeared intact in BM small pre-B cells, about 50% deleted in T1 B cells, and mostly deleted in mature FO B cells in *TSC1* ^*f/f*^-*CD19Cre* mice (Fig 3C). Because Tsc1 is not deleted or inefficiently deleted during early B-cell development, our study does not rule out that TSC1 may play an important role in the development of these cells.

### Differential effects of TSC1 deficiency on mTOR complexes 1 and 2 signaling

**Fig 4:**
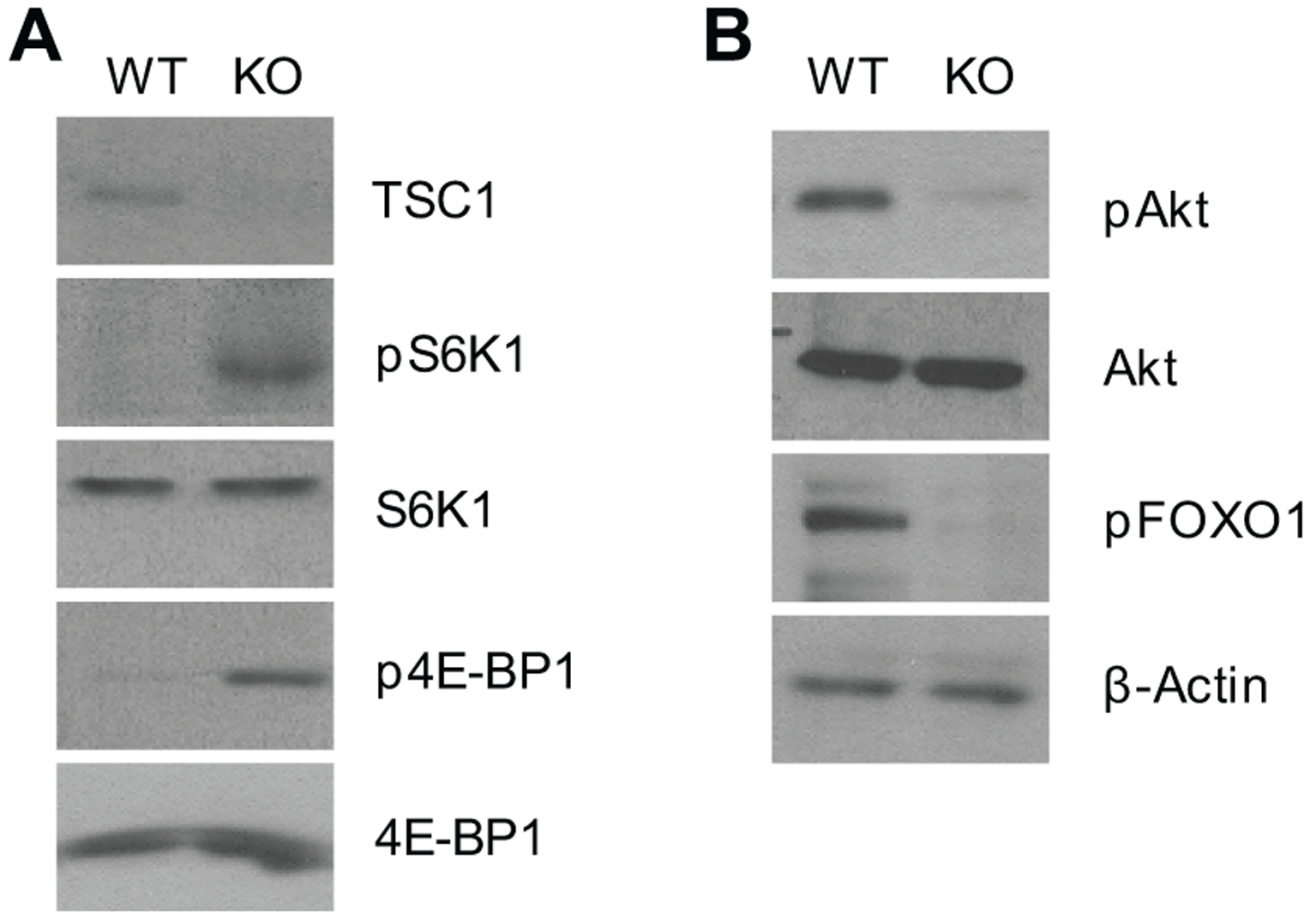
Differential effects of TSC1 deficiency on mTORC1 and mTORC2 signaling in B cells. Sorted CD93^-^B220^high^ mature B cells from WT and TSC1KO splenocytes were lyzed in 1%-NP-40 lysis buffer and subjected to immunoblotting analysis with the indicated antibodies. (A) Enhanced mTORC1 signaling in TSC1KO mature B cells. (B) Decreased mTORC2 signaling in TSC1KO mature B cells. Data shown are representative of three experiments.

To investigate how TSC1 deficiency may affect mTOR signaling in B cells, we sorted mature B cells from the WT and TSC1KO spleens and prepared lysates from sorted B cells for immunoblot analysis (Fig 4). TSC1 was readily detected in WT but not in TSC1KO B cells, indicating effective deletion of the *TSC1* gene in TSC1KO B cells. Phosphorylation of S6K1 at T421/S424 and 4E-BP1 at T37/S46, events that are dependent on mTORC1, was drastically increased in TSC1KO B cells, indicating enhanced mTOR signaling (Fig 4A). In contrast, Akt phosphorylation at Ser473, an mTORC2-mediated event, was substantially decreased in TSC1KO B cells (Fig 4B). Furthermore, Foxo1 phosphorylation at S256, an Akt-mediated event, was also decreased in TSC1KO B cells. Thus, mTORC1 signaling is enhanced in TSC1KO B cells, while mTORC2 signaling and Akt activity were reduced in the absence of TSC1.

### TSC1 in B cells is dispensable for GC formation

**Fig 5:**
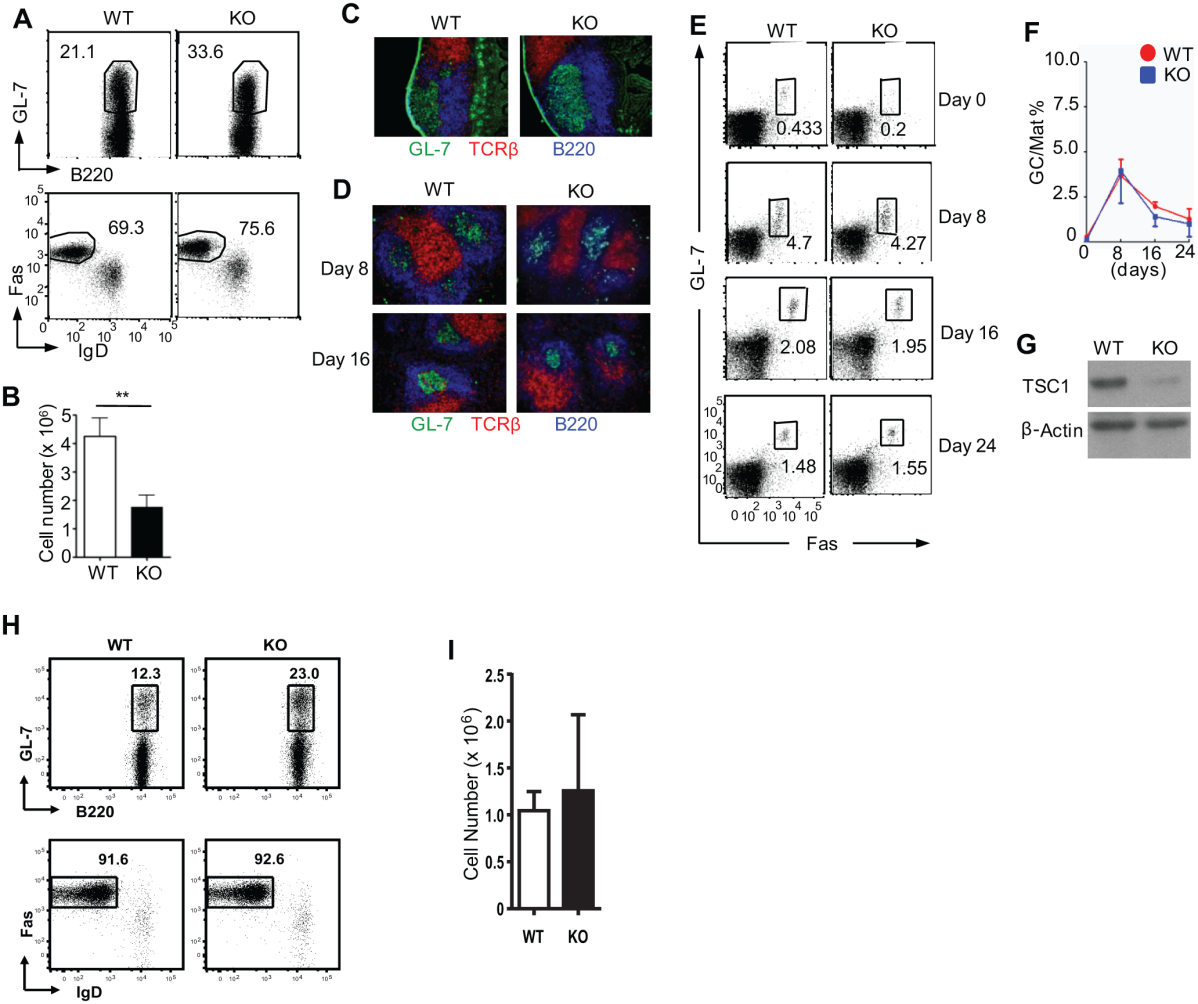
TSC1 in B cells is not required for germinal center formation. (A-C) Assessment of PP GC in WT and TSC1KO mice. (A) GL-7 and Fas staining in live-gated CD93^-^B220^+^ compartment from WT and TSC1KO PPs. (B) Total cell number in PP (n = 7). (C) Architecture of PPs revealed by immunofluorescent microscopy. Frozen thin sections of PPs were probed with B220-AF350 (blue), TCR-PE (red), and GL-7-FITC (green) antibodies. FITC signal was amplified using anti-FITC-AF488 antibody. Images were acquired using a Zeiss Axiovert 200M confocal immunofluorescent microscope. (D-G) Assessment of GC formation in WT and TSC1KO spleens following immunization. Mice were i.p. injected with 20 μg NP_11_-CGG precipitated in alum. Spleens were harvested at days 0, 8, 16, and 24 postimmunization. (D) Detection of GC in the spleen 8 days and 16 days after immunization by immunofluorescence. (E, F) GC B cell frequencies measured by flow cytometry. (E) Representative dot plot of GL-7 and Fas staining in live-gated CD93^-^B220^high^ cells. (F) Percentage of GL-7^+^Fas^+^IgD^-^ GC B cells within the CD93^-^B220^high^ population (n = 6). (G) Detection of TSC1 protein in sorted CD93^-^B220^high^ GL7^+^Fas^+^ GC B cells from WT and TSC1KO splenocytes 8 days after immunization by immunoblotting. (H, I) GC B cells in PP from WT and TSC1KO mice in C57/BL6-129 mixed background. (H) Representative dot plots. (I) GC B cell numbers (n = 4). Data were calculated from four (A-C), three (D-G), or two (H, I) independent experiments. **p< 0.01.

GCs are specialized microenvironments where antigen-activated B cells undergo proliferation, immunoglobulin (Ig) class-switch recombination, somatic hypermutation (SHM), and affinity maturation [9]. Despite extensive studies, however, regulation of GC formation is not fully understood. In this regard, a recent study reported defective GC formation in mice deficient for TSC1 in B cells [46]. To further explore roles for TSC1 in the regulation of GC, we examined Peyer’s patches (PPs), which contain constitutive GCs in WT mice. In contrast to the previous report [46], we were surprised to observe that GC formation was virtually intact in TSC1KO mice (Fig 5A–5C). The frequency of GL-7^+^Fas^+^IgD^-^ GC B cells within B220^+^ PP cells in TSC1KO mice was comparable to or even higher than that in WT mice (Fig 5A), although total cellularity in TSC1KO PPs was about 50% of WT PPs (Fig 5B). In addition, histological analysis of PPs showed similar clusters of GL-7^+^B220^+^ cells between TSC1KO mice and WT mice, indicating the proper structural organization of GCs in PPs of TSC1KO mice (Fig 5C).

To further determine the role of TSC1 in GC formation, we induced GC responses by intraperitoneal immunization with TD antigen, NP_11_-CGG, precipitated in alum [51], and compared the kinetics of GC responses in WT and TSC1KO mice. GCs (clusters of GL-7^+^B220^+^ cells) were readily detected in TSC1KO spleens 8 and 16 days after immunization (Fig 5D). Flow cytometric analyses revealed that frequency of GL-7^+^Fas^+^IgD^-^ GC B cells within B220^high^ mature B cells was similar in WT and TSC1KO spleens during the course of study (Fig 5E and 5F). A substantially lower amount of TSC1 protein in TSC1KO GC B cells than in WT GC B cells indicates that the uncompromised GC responses in TSC1KO mice were not due to the expansion of TSC1-sufficient B cells (Fig 5G). Together, these data indicate that TSC1 expression in B cells is dispensable for constitutive GC formation in PPs and TD GC formation in the spleen.

Our data contradict those finding that TSC1 is crucial for GC formation [46]. Both studies used the *Tsc1* ^*f/f*^ mice provided by the Jackson Lab that were in a C57B6/129-mixed background before being backcrossed to a C57BL/6 background. To examine whether such mixed background might cause differential requirements of TSC1 for GC formation, we examined *TSC1* ^*f/f*^ *-CD19Cre* and *TSC* ^*f/f*^mice in a C57BL/6J/129-mixed background and found similar frequencies of GC B cells in PPs in these mice (Fig 5H and 5I), further supporting that constitutive GC formation was not affected by TSC1 deficiency.

### Effects of TSC1 deficiency on antibody responses to a T-cell-dependent antigen

**Fig 6:**
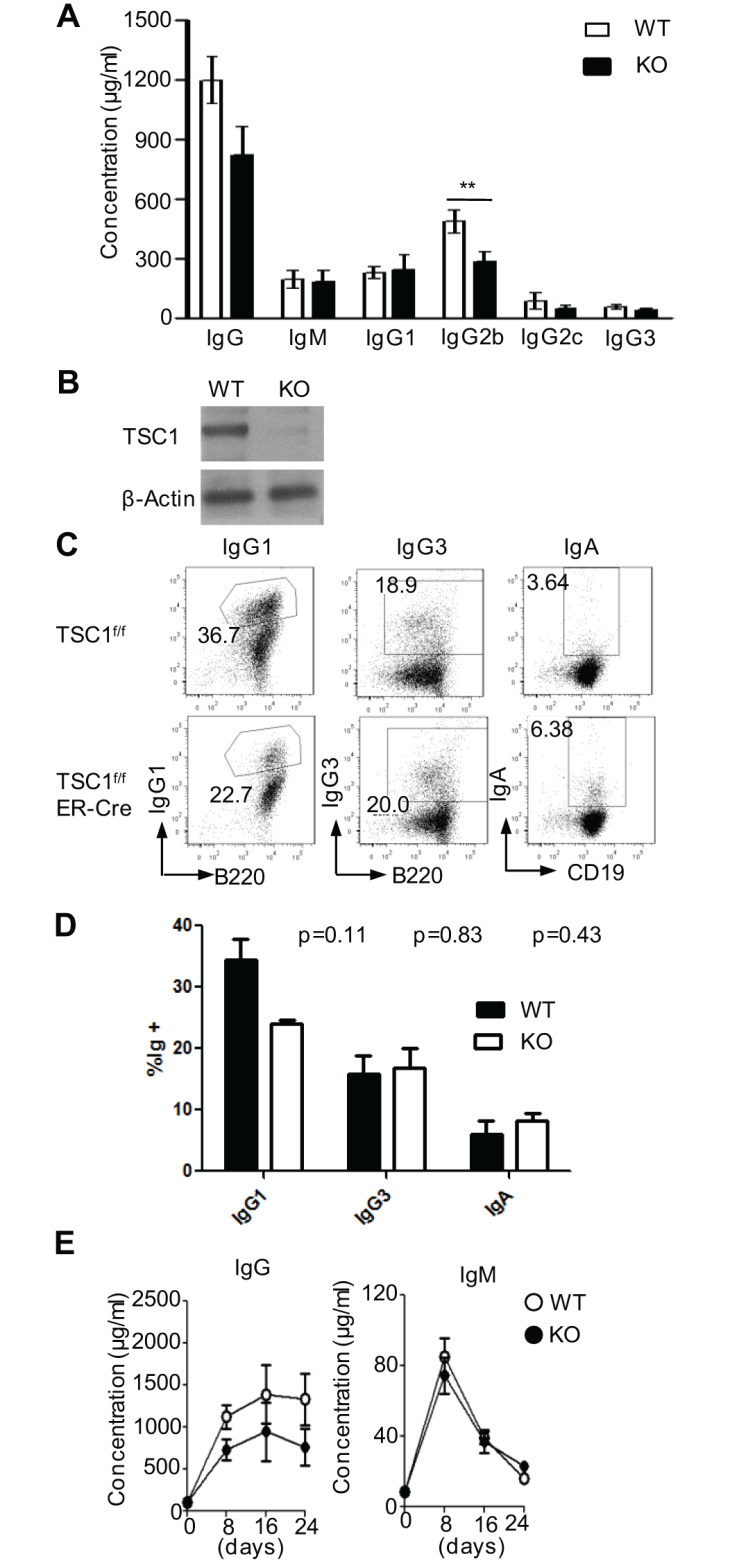
Effects of TSC1 deficiency on *Ig* CSR and serum antibody responses. (A) Serum Ig levels in unimmunized WT and TSC1KO mice (n = 9). (B) TSC1 protein levels in sorted B220^+^ B cells from *Tsc1* ^*f/f*^ *ERcre* ^-^ and *Tsc1* ^*f/f*^ *ERcre* ^+^ mice after tamoxifen injections. (C, D) CSR is not significantly reduced in TSC1-deficient B cells. B cells from *Tsc1* ^*f/f*^ *ERcre* ^-^ and *Tsc1* ^*f/f*^ *ERcre* ^+^ mice after multiple tamoxifen injections were purified and induced to CSR to the indicated isotype for 4 days and analyzed by flow cytometry. (C) Representative dot plots from cells induced to switch to IgG1 (n = 3), IgG3 (n = 2), and IgA (n = 3). (D) Quantification of data from (C). Shown are means ± SD. IgG1 and IgA samples were subjected to 2-tailed paired Student’s *t* test, and IgG3 was subjected to 2-tailed unpaired Student’s *t* test. (E) Serum NIP_25_-specific IgM and IgG in the same mice immunized with NP_11_-CGG in alum, as described in Fig 5D–5G (n = 6). *p< 0.05; **p< 0.01.

The reduction of mature B cells but apparently normal GC induction in TSC1KO mice led us to investigate antibody response in TSC1KO mice as well. We first measured serum Ig levels in unimmunized WT and TSC1KO mice by ELISA. The TSC1KO serum contained similar levels of total IgM and IgG as well as most IgG subtypes to WT controls, with the exception of IgG2b, which was reduced about 40% in TSC1KO mice (Fig 6A). To further examine whether TSC1 regulates *Ig* CSR, we deleted TSC1 in mature B cells by injection of tamoxifen into *Tsc1* ^*f/f*^-*ERcre* ^+^ and *Tsc1* ^*f/f*^-*ERcre* ^-^ mice. Western Blot Analysis showed efficient ablation of TSC1 in sorted *Tsc1* ^*f/f*^ *-ERcre* ^+^ B cells after tamoxifen injection (Fig 6B). To induce CSR in B cells in vitro, we cultured WT and TSC1-deleted splenic B220^+^ B cells in the presence of LPS and cytokines, and then analyzed them for surface IgG1, IgG3, and IgA expression by flow cytometry. Under these ex vivo switching conditions, switching to IgG3 and IgA was comparable between WT and TSC1-deleted B cells. However, switching to IgG1 in TSC1-deleted B cells appeared reduced compared to WT B cells (Fig 6C and 6D). These observations show that although TSC1 expression in B cells is not essential for the *Ig* CSR or the production of serum IgG and IgA, its deficiency causes mild reduction of *Ig* CSR and specific serum IgG.

We further examined the effect of TSC1 deficiency on the induction of TD antibody responses following NP_11_-CGG immunization. As shown in Fig 6E, NP-specific (NIP_25_-binding) IgG titers were slightly decreased in TSC1KO mice but statistically insignificant (*p*>0.05), while no obvious difference was observed in serum NIP IgM Ab between WT and TSC1KO mice at 8, 16, and 24 days postimmunization. Our data suggest that TSC1 deficiency does not obviously affect the induction of IgM antibody responses and only mildly impacts IgG antibody responses to TD antigens.

## Discussion

In this report we demonstrated that TSC1 differentially controls mTORC1 and mTORC2 signaling in B cells and that TSC1 deficiency in B cells results in accumulation of T1 B cells but increasingly impaired maturation/maintenance of T2 B cells and the FO and MZ B cell compartments. Despite significant effects on B-cell maturation, TSC1 deficiency in B cells did not significantly change GC responses, CSR, or serum antibody responses against immunization with TD antigen as well as in the steady state.

Our data indicate that TSC1 is important for B-cell development and/or maintenance at multiple stages. We have demonstrated that TSC1 deficiency in B cells leads to an increase of T1 B cells but a decrease of mature B cells (Fig 2). This increase of T1 B cell numbers in our study is consistent with the previous report by Benhamron and Tirosh [46]. Using WT and TSC1KO-mixed BM chimeric mice, we showed that TSC1KO T1 B cells were overrepresented, but T2 B cells were underrepresented in the same animals (Fig 3B), revealing defective T1 to T2 maturation in the absence of TSC1, which was not clearly reported by Benhamron and Tirosh [46]. In addition to promoting T1 to T2 maturation, our data also suggest that TSC1 plays additional roles for B-cell maturation or maintenance in later stages of B-cell development. Evidence supporting a role for TSC1 in B cells beyond the T1 to T2 transition includes near normal number of T2 B cells but a severe decrease of both FO and MZ B cells in the spleens of TSC1KO mice (Fig 2H, 2K and 2L). While developmental blockade is the likely explanation for the decreases of B cells in TSC1KO mice, it is also possible that TSC1 may promote B cell homeostatic proliferation and/or survival.

GC reaction is critical to mount effective humoral immune responses against pathogens. In GCs, antigen-specific, activated B cells undergo clonal expansion and SHM in the *Ig* loci as well as affinity maturation [6, 9]. SHM introduces point mutations in the variable region of *Ig* genes in B cells. Subsequently, B cells expressing B-cell antigen receptors with high affinity for its cognate antigen preferentially expand in the GCs, the process underlies the affinity maturation [52]. We have revealed constitutive GC formations in PPs of unimmunized TSC1KO mice as well as virtually intact splenic GC formations in TSC1KO mice following immunization (Fig 5C and 5D). In addition, TSC1 deficiency causes only a mild decrease of IgG response without obviously affecting the overall IgM responses to a TD antigen (Fig 6E). Thus, TSC1 deficiency does not obviously affect GC formation. Of note, our data are inconsistent with Benhamron and Tirosh’s study in which they observed a severe defect of GC formation in TSC1KO mice [46]. In C57BL/6/129 mixed-background TSC1KO mice, we did not observe obvious impairment of constitutive GC B cell formation in PPs. However, our data did not firmly rule out that inducible GC B cell formation following immunization could be affected in these mice. At present, the reasons that lead to the discrepancies between Benhamron and Tirosh’s study and ours are unclear.

Using sorted mature B cells from the spleen, we have demonstrated that TSC1 inhibits mTORC1 while promoting mTORC2 signaling in B cells (Fig 4). This observation is consistent with the role of TSC1 in the control of mTOR signaling in T cells, dendritic cells, macrophages, and mast cells [11–14, 37]. In TSC1KO B cells, mTORC2/Akt activities, reflected by Akt phosphorylation at Ser473 and Foxo1 phosphorylation, were decreased. Deficiency of mTORC2 due to Rictor deficiency results in impaired generation of FO and MZ B cells [26]. Additional studies have indicated that Akt promotes MZ B cell generation in the spleen. Decreased Akt activity leads to reduction of MZ B cells, while increased Akt activity caused by PTEN deficiency results in an increase of MZ B cells [53, 54]. Furthermore, deficiency of Foxo1, which is suppressed by Akt-mediated phosphorylation, rescues MZ B cell defects caused by CD19 deficiency [54, 55]. Thus, it is plausible that decreased Akt activity may contribute to the decrease of MZ B cells in TSC1KO mice. Given that phenotypes of abnormal B-cell development in TSC1KO mice appear more profound than those in Akt-deficient mice, the decreased Akt activity in TSC1KO B cells cannot be solely responsible for the abnormal B-cell development in TSC1KO mice. Along with this hypothesis, accumulation and reduction of T1 and T2 B cells, respectively, in TSC1KO mice must be caused by abnormalities other than the decreased Akt activity in TSC1KO B cells inasmuch as Akt deficiency has no impact on the development of T1 and T2 B cells [53]. Future studies should determine the mechanisms involved and the contribution of dysregulated mTORC1/2 signaling to the abnormal phenotypes in TSC1KO B cells.
